# Supplementary material for: Characterizing the postmortem human bone microbiome from surface-decomposed remains
Source: PLoS One. 2020 Jul 8;15(7):e0218636. doi: 10.1371/journal.pone.0218636 (PMC7343130; doi:10.1371/journal.pone.0218636)
Supplement: S1 Table — (DOCX) [file pone.0218636.s001.docx]

Table S1: Donor Information for the three individuals placed at ARF 2009

| Individual | Ancestry | Sex | Weight (kg) | Medical History | Date of Placement | Date of Collection | Length of Placement |
| --- | --- | --- | --- | --- | --- | --- | --- |
| A | European | Male | 104.3 | Diabetes, Alcoholism, Substance abuse | 01/29/09 | 02/17/10 | 13 months |
| B | European | Male | 80.3 | High cholesterol, Arthritis | 11/20/09 | 03/18/11 | 16 months |
| C | European | Male | 127.0 | Diabetes, Cardiac | 04/24/09 | 03/18/11 | 23 months |
