# Supplementary material for: Characterizing the postmortem human bone microbiome from surface-decomposed remains
Source: PLoS One. 2020 Jul 8;15(7):e0218636. doi: 10.1371/journal.pone.0218636 (PMC7343130; doi:10.1371/journal.pone.0218636)
Supplement: S2 Table — (DOCX) [file pone.0218636.s002.docx]

Table S2: Bones sampled from each of the three individuals [1]

| **Bone Type** | **Sample Quantity per Individual** |
| --- | --- |
| **Skull** |  |
| Frontal | 1 |
| Temporal | 1 |
| Parietal | 1 |
| Occipital | 1 |
| Maxilla | 1 |
| Mandible | 1 |
| **Teeth** |  |
| Maxillary lateral incisor  Maxillary canine | 1 |
| Maxillary 1^st^ premolar  Maxillary molar | 1 |
| Mandibular lateral incisor  Mandibular canine | 1 |
| Mandibular 1^st^ premolar | 1 |
| **Trunk** |  |
| Cervical vertebra | 1 |
| Thoracic vertebra | 1 |
| Lumbar vertebra | 1 |
| 1^st^ Rib | 1 |
| Middle rib | 1 |
| 12^th^ Rib | 1 |
| Sternum | 1 |
| Sacrum | 1 |
| Clavicle | 1 |
| Scapula | 1 |
| Ilium | 1 |
| Ischium | 1 |
| Pubis | 1 |
| **Leg** |  |
| Femur | 1 |
| Tibia | 1 |
| Fibula | 1 |
| Patella | 1 |
| **Arm** |  |
| Humerus | 1 |
| Radius | 1 |
| Ulna | 1 |
| **Hand** |  |
| Metacarpals 1–5 | 5 |
| 1^st^ Proximal phalanx | 1 |
| 1^st^ Distal phalanx | 1 |
| Capitate | 1 |
| **Foot** |  |
| Metatarsals 1–5 | 5 |
| 1^st^ Proximal phalanx | 1 |
| 1^st^ Distal phalanx | 1 |
| Calcaneus | 1 |
| Talus | 1 |
| Navicular | 1 |
| Cuboid | 1 |
| Medial Cuneiform | 1 |
| Intermediate Cuneiform | 1 |
| Lateral Cuneiform | 1 |
| **Total** | 55 |
