# Supplementary material for: Characterizing the postmortem human bone microbiome from surface-decomposed remains
Source: PLoS One. 2020 Jul 8;15(7):e0218636. doi: 10.1371/journal.pone.0218636 (PMC7343130; doi:10.1371/journal.pone.0218636)
Supplement: S3 Table — Data was separated by individual, and the mean and standard deviation was computed by body region. Significance levels are represented by asterisks (*p < 0.05; **p < 0.01); multiple comparison tests by individual were only conducted using Inverse Simpson indices. Lower case letters in parenthesis refer to body regions. A body region with an exponent corresponding to another body region indicates that those two regions have significantly different diversity indices. (DOCX) [file pone.0218636.s003.docx]

Table S3: Bacterial alpha diversity metrics including observed richness and diversity (Inverse Simpson). Data was separated by individual, and the mean and standard deviation was computed by body region. Significance levels are represented by asterisks (**p* < 0.05; ***p* < 0.01); multiple comparison tests by individual were only conducted using Inverse Simpson indices. Lower case letters in parenthesis refer to body regions. A body region with an exponent corresponding to another body region indicates that those two regions have significantly different diversity indices.

| **Individual** | **Body Region** | **Inverse Simpson (mean)** | **Sd. Dev.** | **Richness (mean)** | **Sd. Dev.** |
| --- | --- | --- | --- | --- | --- |
| A | Arm (a) | 35.9 | 6.6 | 309.1 | 1100.4 |
| A | Foot (b) | 24.2 | 14.0 | 420.1 | 1045.7 |
| A | Hand (c) | 57.3 | 35.1 | 332.9 | 1509.6 |
| A | Leg (d) | 36.5 | 21.3 | 310.1 | 1252.5 |
| A | Lower trunk (e) | 23.6 | 30.3 | 232.8 | 1403.3 |
| A | Skull (f) | 34.3 | 48.9 | 484.0 | 1273.9 |
| A | Tooth (g)^c*^ | 9.2 | 6.4 | 168.3 | 699.5 |
| A | Upper trunk (h) | 26.2 | 25.9 | 327.7 | 1168.9 |
| B | Arm (a)^c*,h*^ | 19.3 | 3.8 | 213.9 | 757.9 |
| B | Foot (b)^c**,g*^ | 63.0 | 49.0 | 970.4 | 1915.6 |
| B | Hand (c)^a*,b**,d*,e*,f*,h*^ | 6.1 | 2.1 | 203.5 | 478.0 |
| B | Leg (d)^c*^ | 37.2 | 31.4 | 368.7 | 789.5 |
| B | Lower trunk (e)^a*,c*,f*,g*^ | 59.0 | 12.0 | 276.5 | 1363.4 |
| B | Skull (f)^c*,e*^ | 28.3 | 10.5 | 235.2 | 807.1 |
| B | Tooth (g)^b*,e*,h*^ | 15.9 | 11.0 | 156.4 | 397.5 |
| B | Upper trunk (h)^c*,g*^ | 61.3 | 27.9 | 653.9 | 1848.9 |
| C | Arm (a) | 25.5 | 9.4 | 410.4 | 944.0 |
| C | Foot (b)^g*^ | 44.7 | 24.5 | 335.1 | 1078.0 |
| C | Hand (c) | 34.3 | 14.4 | 212.1 | 979.9 |
| C | Leg (d) | 41.3 | 18.2 | 569.4 | 1348.6 |
| C | Lower trunk (e) | 91.0 | 43.1 | 533.3 | 1824.3 |
| C | Skull (f) | 45.8 | 36.5 | 689.5 | 1400.9 |
| C | Tooth (g)^b*,h*^ | 14.5 | 7.4 | 422.9 | 1005.3 |
| C | Upper trunk (h)^g*^ | 79.8 | 47.8 | 574.5 | 2302.5 |
