# Supplementary material for: Characterizing the postmortem human bone microbiome from surface-decomposed remains
Source: PLoS One. 2020 Jul 8;15(7):e0218636. doi: 10.1371/journal.pone.0218636 (PMC7343130; doi:10.1371/journal.pone.0218636)
Supplement: S1 Fig — The minimum library size was 48,288 reads, while the mean library size was 92,334.6 reads; the maximum library was 150,228 reads. (DOCX) [file pone.0218636.s004.docx]

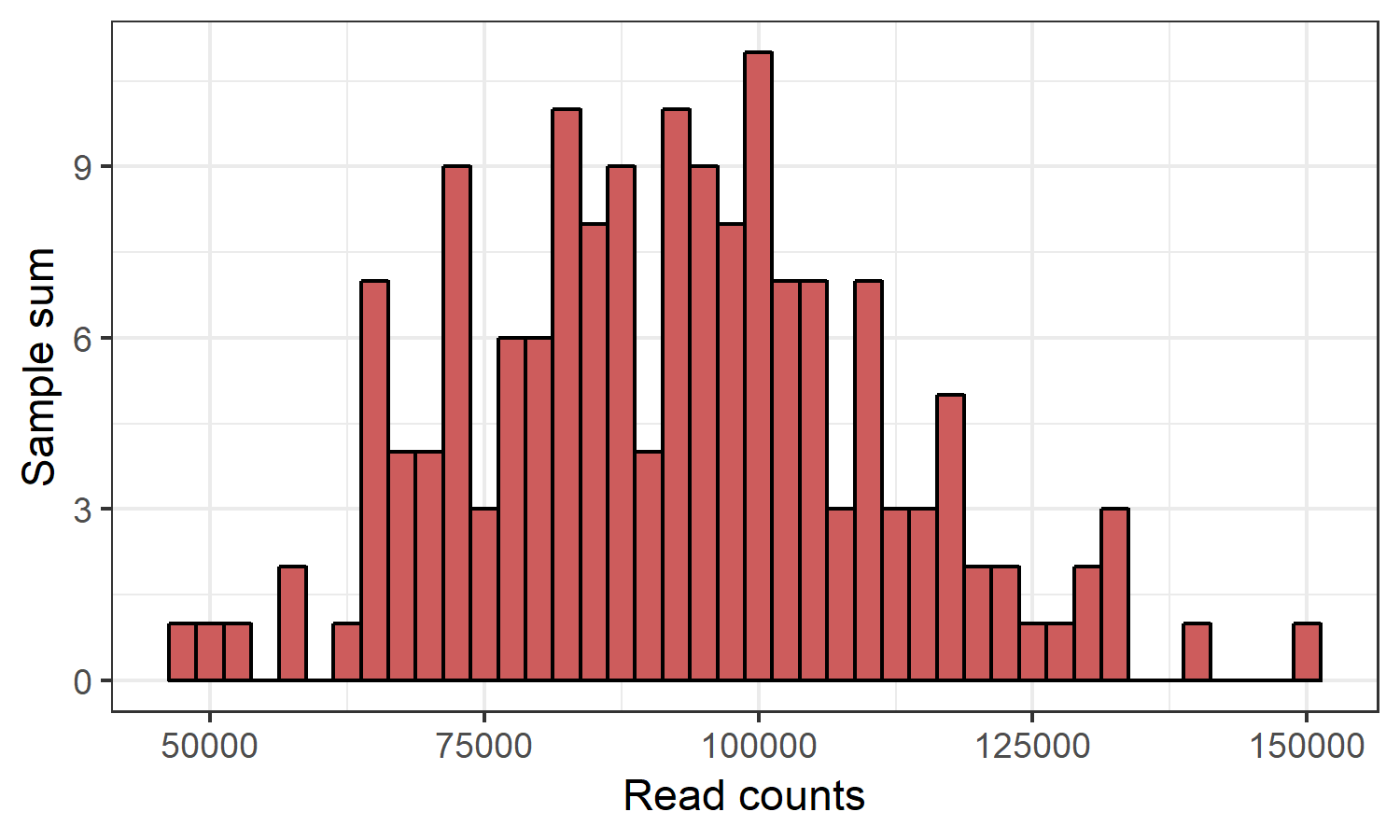


Figure S1: 16S rRNA targeted metagenomics read distribution. The minimum library size was 48,288 reads, while the mean library size was 92,334.6 reads; the maximum library was 150,228 reads.
