# Supplementary material for: Characterizing the postmortem human bone microbiome from surface-decomposed remains
Source: PLoS One. 2020 Jul 8;15(7):e0218636. doi: 10.1371/journal.pone.0218636 (PMC7343130; doi:10.1371/journal.pone.0218636)
Supplement: S2 Fig — The minimum library size was 5,368 reads; the mean library size was 19,853 reads, and the maximum library size was 40,977 reads. (DOCX) [file pone.0218636.s005.docx]

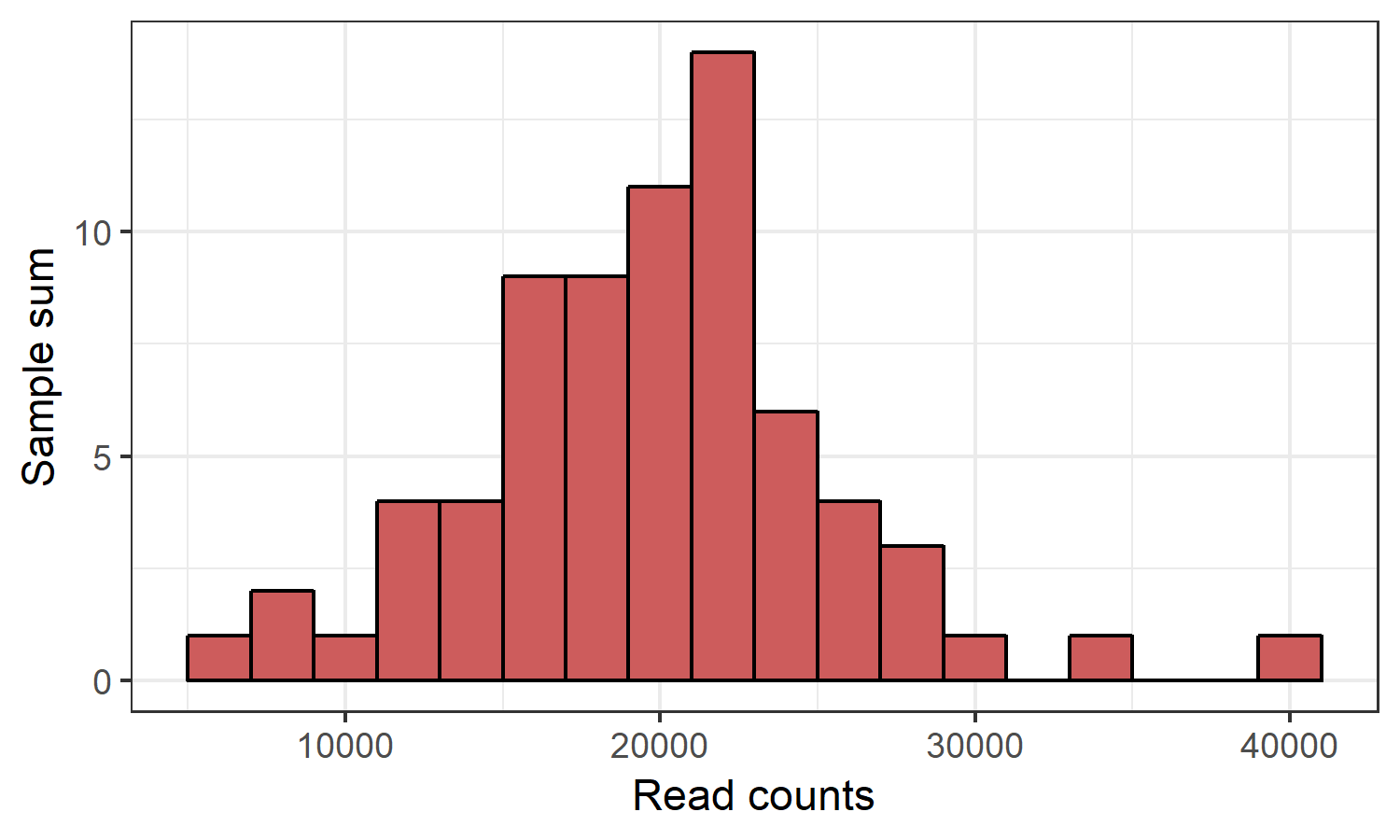


Figure S2: 18S rRNA targeted metagenomics read distribution. The minimum library size was 5,368 reads; the mean library size was 19,853 reads, and the maximum library size was 40,977 reads.
