# Supplementary material for: Characterizing the postmortem human bone microbiome from surface-decomposed remains
Source: PLoS One. 2020 Jul 8;15(7):e0218636. doi: 10.1371/journal.pone.0218636 (PMC7343130; doi:10.1371/journal.pone.0218636)
Supplement: S3 Fig — **Indicate significance below an alpha value of 0.01. No significance is denoted by “ns”. (DOCX) [file pone.0218636.s006.docx]

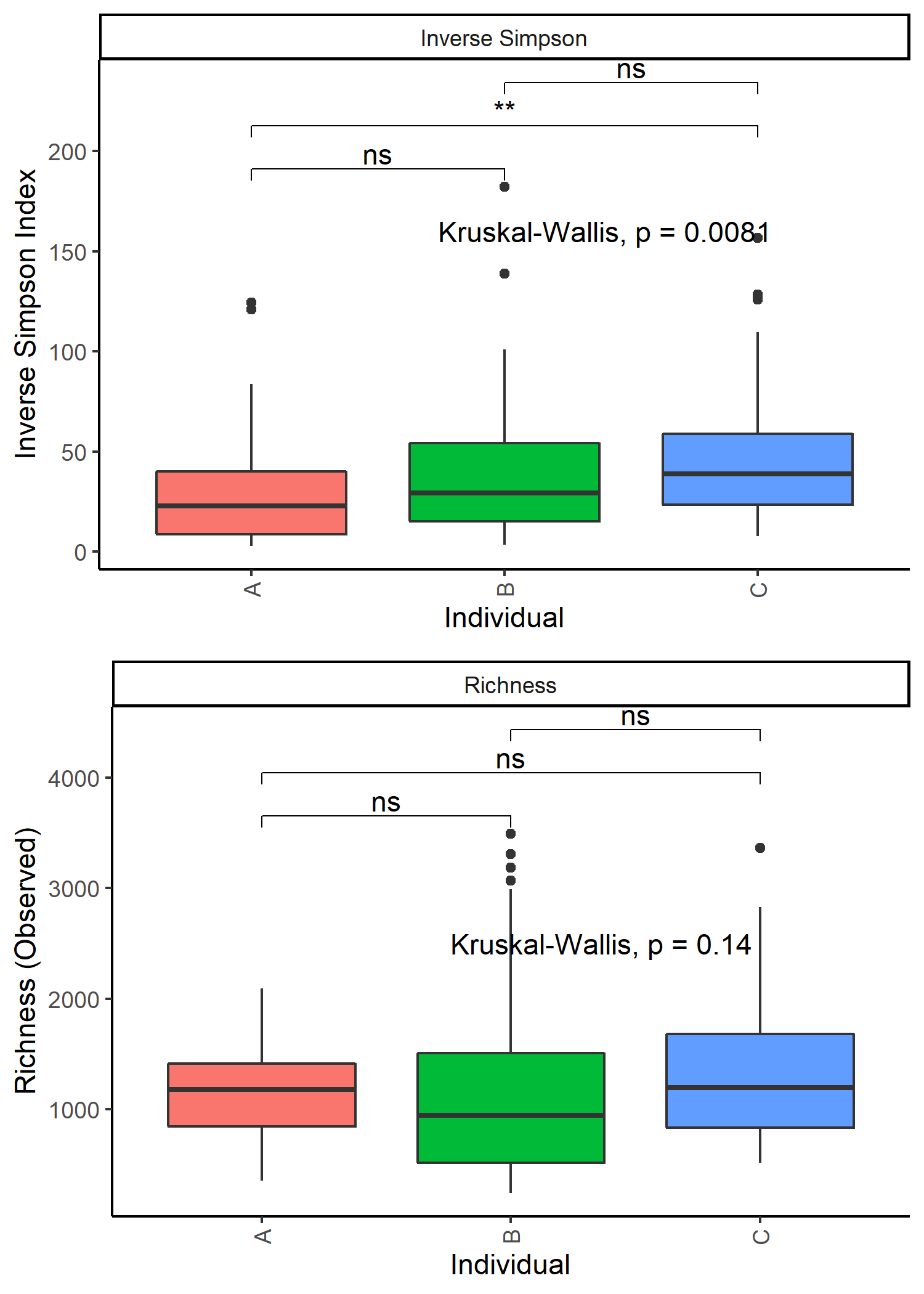


Figure S3: Bacterial alpha diversity (Inverse Simpson) and richness (observed) by individual. **Indicate significance below an alpha value of 0.01. No significance is denoted by “ns”.
