# Supplementary material for: Characterizing the postmortem human bone microbiome from surface-decomposed remains
Source: PLoS One. 2020 Jul 8;15(7):e0218636. doi: 10.1371/journal.pone.0218636 (PMC7343130; doi:10.1371/journal.pone.0218636)
Supplement: S4 Fig — Individuals (“A”, “B”, and “C”) were assessed independently. (DOCX) [file pone.0218636.s007.docx]

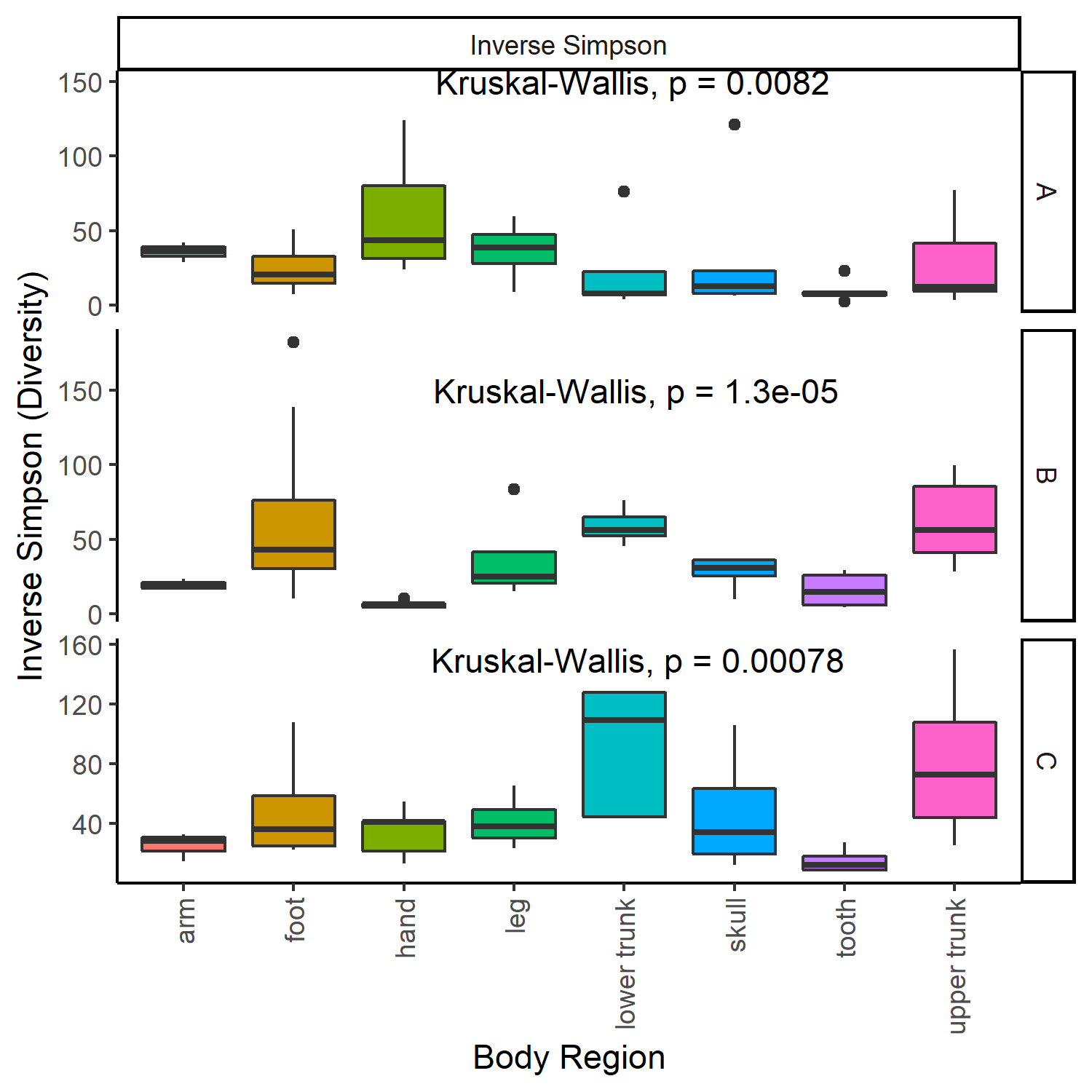


Figure S4: Inverse Simpson (diversity) calculated from the bacterial dataset. Individuals (“A”, “B”, and “C”) were assessed independently.
