# Supplementary figures and images for: Characterizing the postmortem human bone microbiome from surface-decomposed remains
Source: PLoS One. 2020 Jul 8;15(7):e0218636. doi: 10.1371/journal.pone.0218636 (PMC7343130; doi:10.1371/journal.pone.0218636)

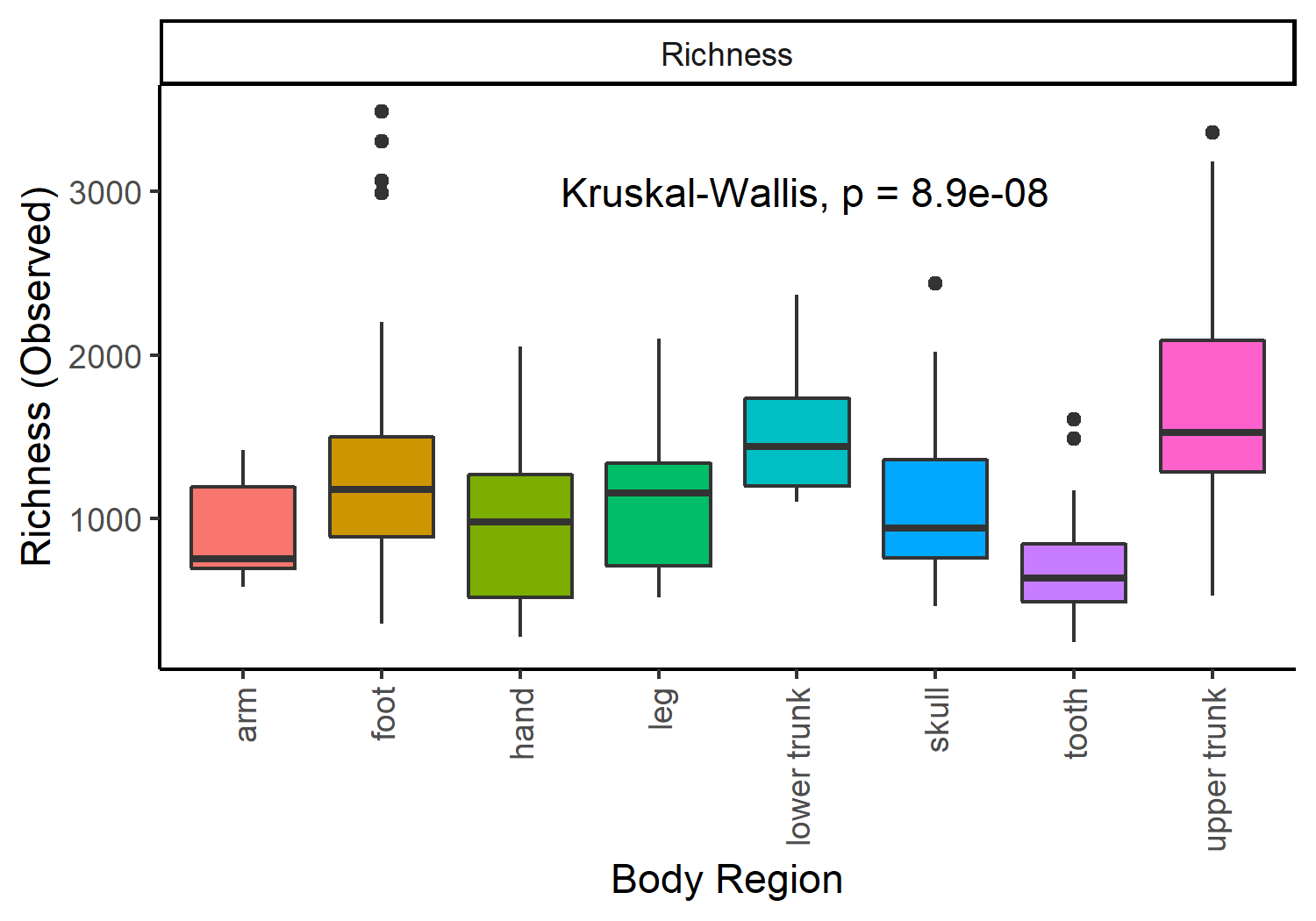


Figure S5: Richness (observed) calculated from the bacterial dataset; individuals were combined.

Supplement: S5 Fig — (DOCX) [file pone.0218636.s008.docx]
