# Supplementary material for: Characterizing the postmortem human bone microbiome from surface-decomposed remains
Source: PLoS One. 2020 Jul 8;15(7):e0218636. doi: 10.1371/journal.pone.0218636 (PMC7343130; doi:10.1371/journal.pone.0218636)
Supplement: S6 Fig — SIMPER results by individual (A: n = 53, B: n = 55, C: n = 54). Results include only those OTUs that demonstrated a significant difference by individual following SIMPER. All OTUs of a given genus are not represented here. The x-axis contains information on the taxonomic identification of these OTUs at the family level. (DOCX) [file pone.0218636.s009.docx]

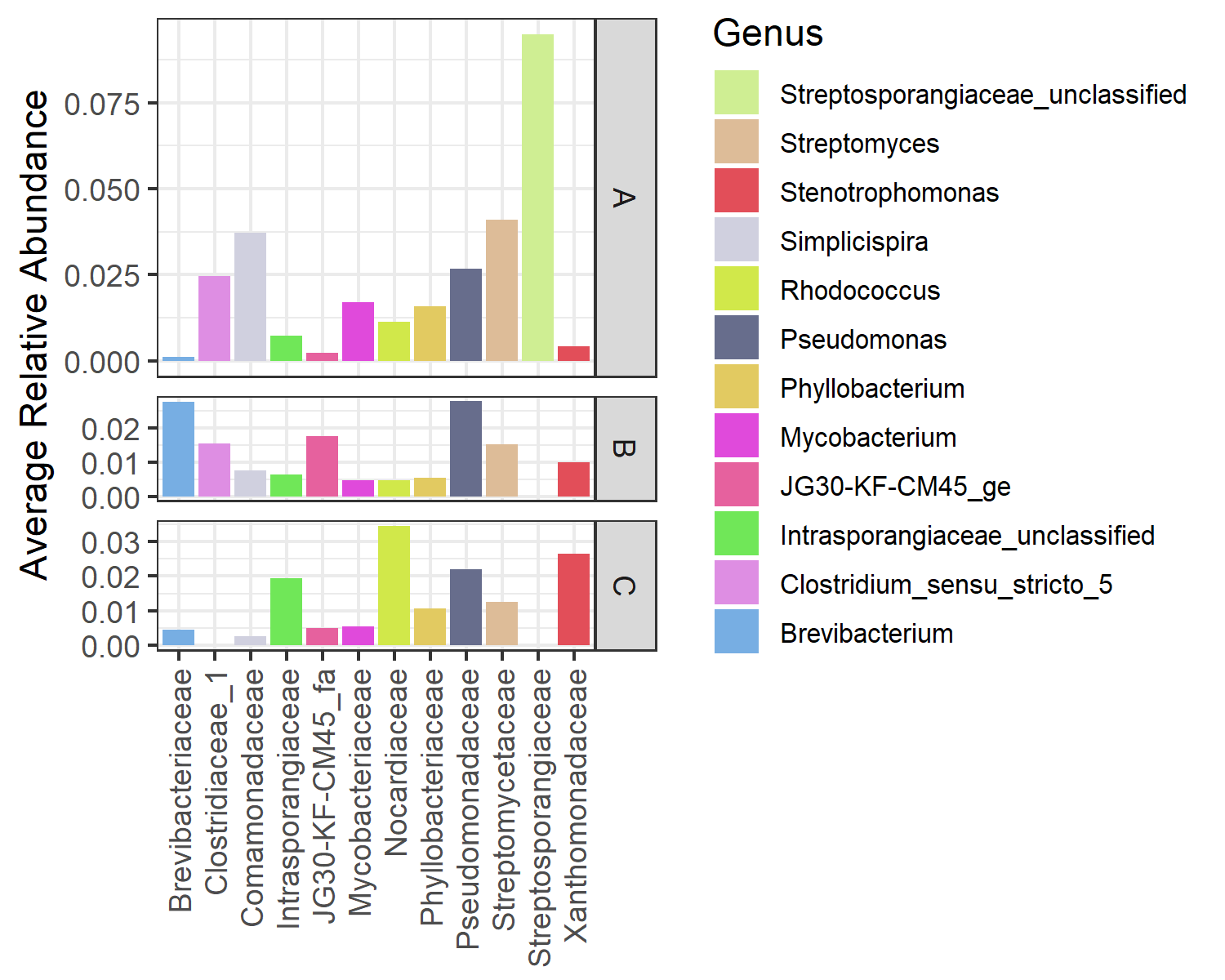


Figure S6: SIMPER results by individual (A: n=53, B: n=55, C: n=54). Results include only those OTUs that demonstrated a significant difference by individual following SIMPER. All OTUs of a given genus are not represented here. The x-axis contains information on the taxonomic identification of these OTUs at the family level.
