# Supplementary material for: Characterizing the postmortem human bone microbiome from surface-decomposed remains
Source: PLoS One. 2020 Jul 8;15(7):e0218636. doi: 10.1371/journal.pone.0218636 (PMC7343130; doi:10.1371/journal.pone.0218636)
Supplement: S7 Fig — (A) Bacterial OTUs important for predicting human DNA concentrations. Importance, as a value between zero to one, is represented in color. Human DNA concentrations greater than 400 ng gbp-1 are labeled by body region. Abundance refers to relative abundance by bone sample, represented by values zero to one. (B) Model accuracy as a function of test values versus predicted values. The solid red line represents the modeled data; the dotted line represents an expected model if 100% accuracy. (DOCX) [file pone.0218636.s010.docx]

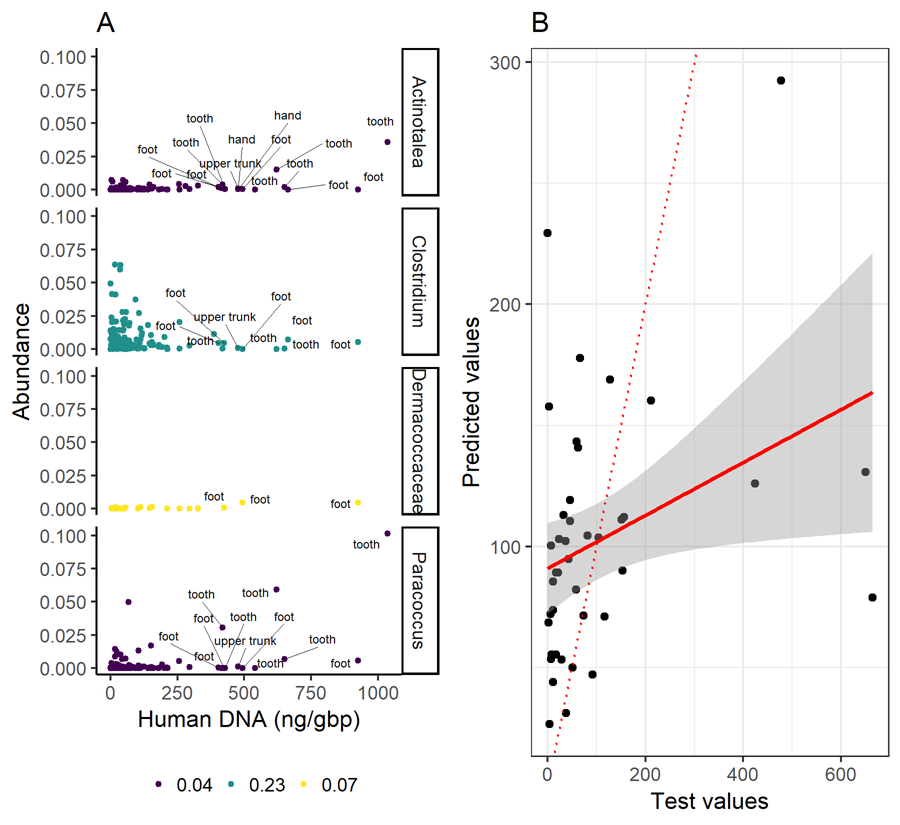


Figure S7**:** Random forest regression. (A) Bacterial OTUs important for predicting human DNA concentrations. Importance, as a value between zero to one, is represented in color. Human DNA concentrations greater than 400 ng gbp^-1^ are labeled by body region. Abundance refers to relative abundance by bone sample, represented by values zero to one. (B) Model accuracy as a function of test values versus predicted values. The solid red line represents the modeled data; the dotted line represents an expected model if 100% accuracy.
