# Supplementary material for: Characterizing the postmortem human bone microbiome from surface-decomposed remains
Source: PLoS One. 2020 Jul 8;15(7):e0218636. doi: 10.1371/journal.pone.0218636 (PMC7343130; doi:10.1371/journal.pone.0218636)
Supplement: S8 Fig — The label “B” refers to individual B. (DOCX) [file pone.0218636.s011.docx]

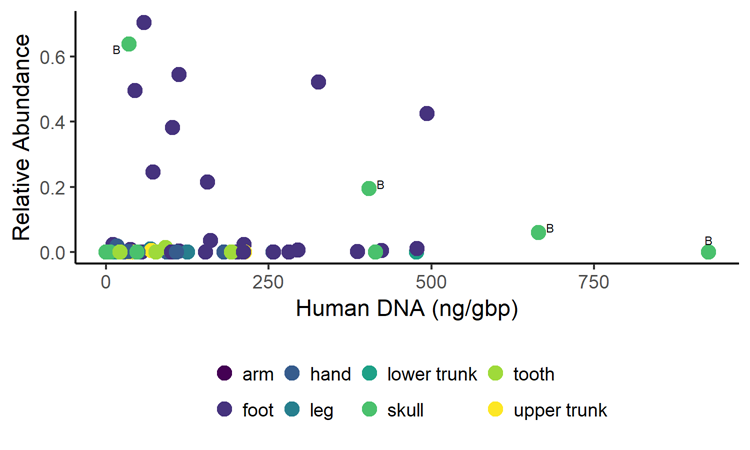


Figure S8: Relative abundance of the unclassified Saccharomycetales OTU plotted against human DNA concentration. The label “B” refers to individual B.
