# Supplementary material for: Characterizing the postmortem human bone microbiome from surface-decomposed remains
Source: PLoS One. 2020 Jul 8;15(7):e0218636. doi: 10.1371/journal.pone.0218636 (PMC7343130; doi:10.1371/journal.pone.0218636)
Supplement: S9 Fig — Donors are labeled “A”, “B”, “C”, and “ADD” refers to accumulated degree days, an indicator of both time and temperature. Data obtained from NOAA (https://www.noaa.gov/). (DOCX) [file pone.0218636.s012.docx]

Figure S9: Changes in temperature and precipitation for the duration of deposition of each donor. Donors are labeled “A”, “B”, “C”, and “ADD” refers to accumulated degree days, an indicator of both time and temperature. Data obtained from NOAA (<https://www.noaa.gov/>).
