# Supplementary material for: Characterizing the postmortem human bone microbiome from surface-decomposed remains
Source: PLoS One. 2020 Jul 8;15(7):e0218636. doi: 10.1371/journal.pone.0218636 (PMC7343130; doi:10.1371/journal.pone.0218636)
Supplement: S10 Fig — Relative abundance was averaged by bone type, combining results from three individuals (n = 3). Only taxa with average relative abundances greater than 1% are shown. (DOCX) [file pone.0218636.s013.docx]

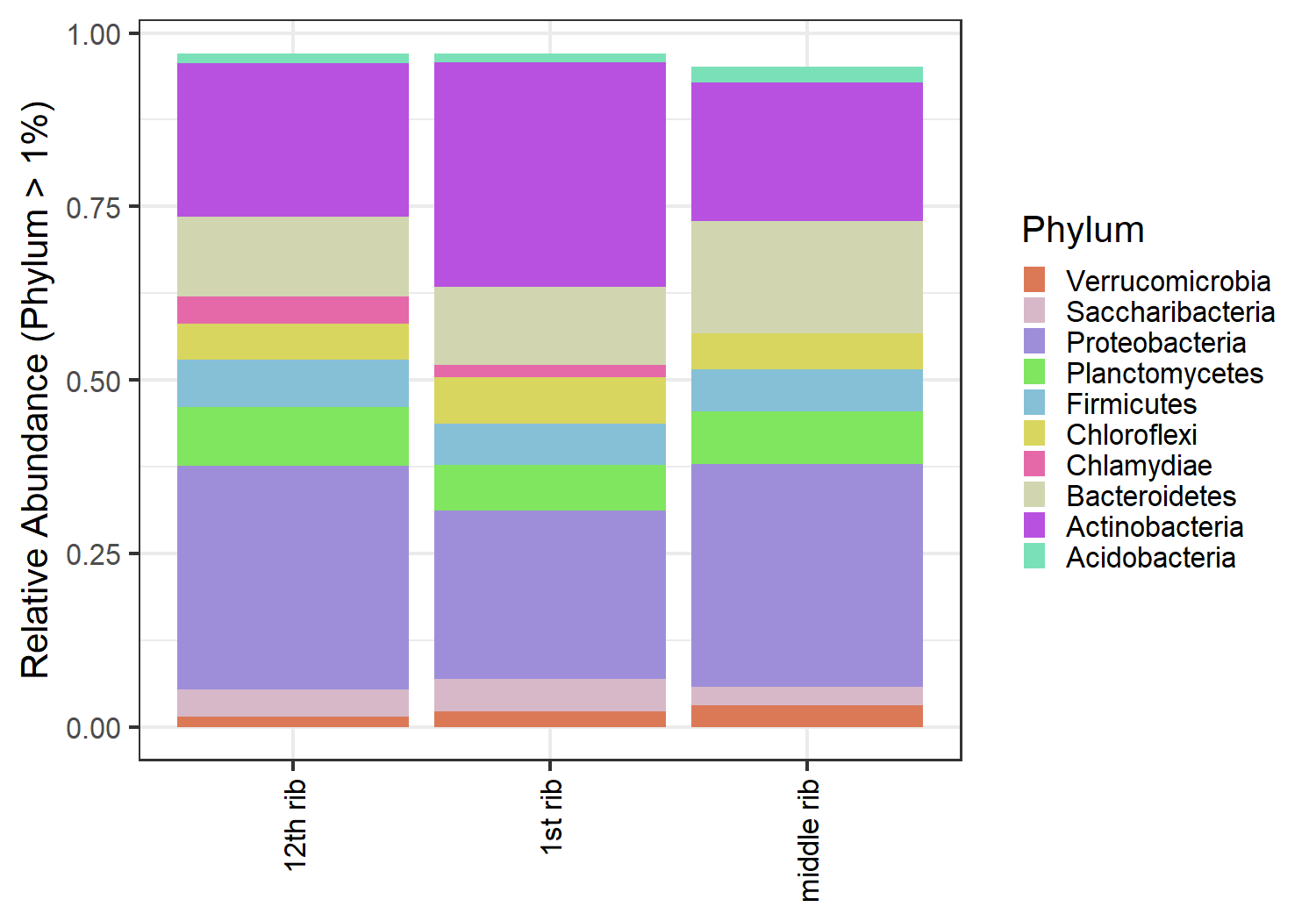


Figure S10: Phylum-level bacterial community membership in human rib samples. Relative abundance was averaged by bone type, combining results from three individuals (n = 3). Only taxa with average relative abundances greater than 1% are shown.
