# Supplementary material for: Characterizing the postmortem human bone microbiome from surface-decomposed remains
Source: PLoS One. 2020 Jul 8;15(7):e0218636. doi: 10.1371/journal.pone.0218636 (PMC7343130; doi:10.1371/journal.pone.0218636)
Supplement: S11 Fig — Bacterial community phylum-level contributions visualized by individual (“A”, “B”, and “C”) and body region (arm, foot, hand, leg, lower trunk, skull, tooth, upper trunk). Only relative abundances greater than 1% are displayed. (DOCX) [file pone.0218636.s014.docx]

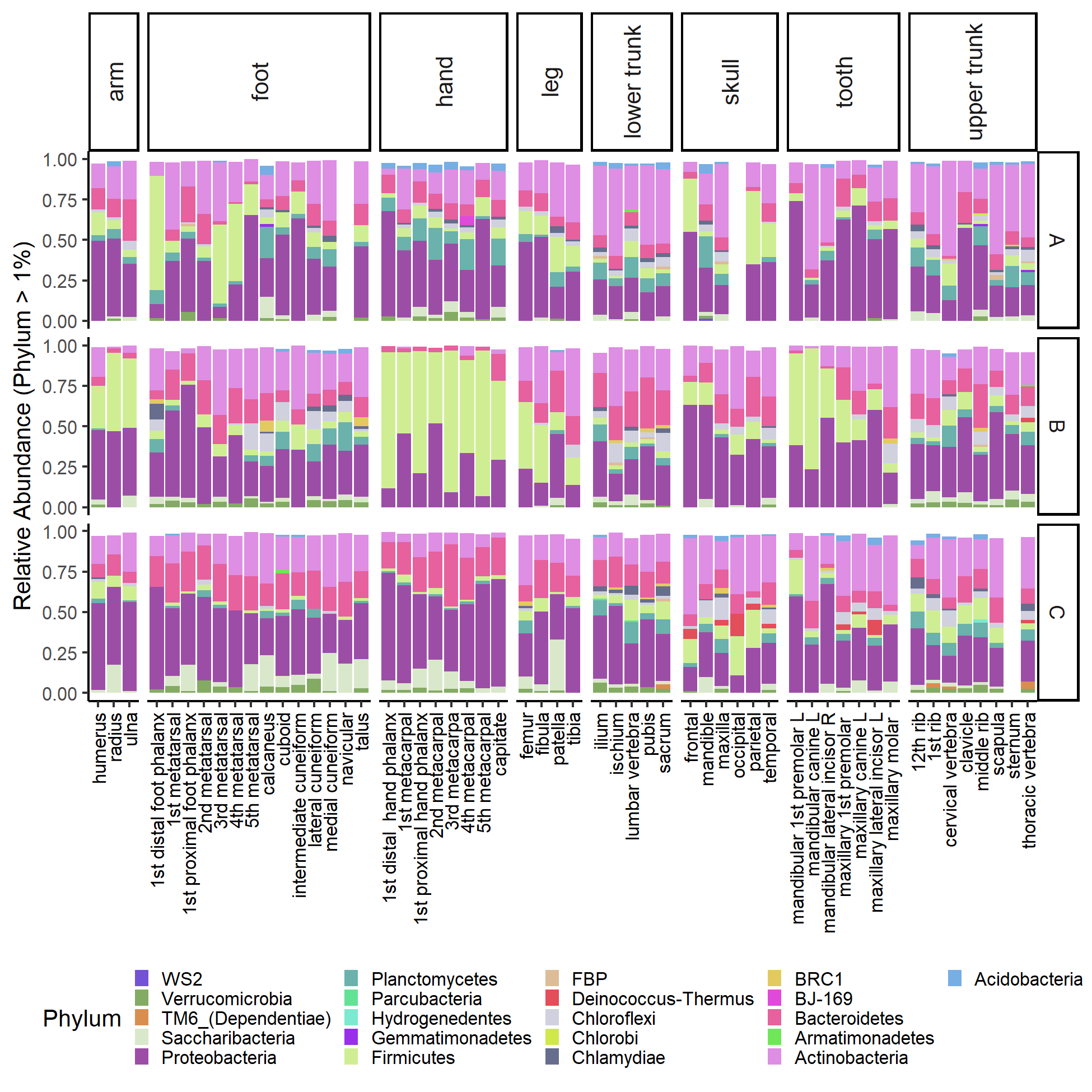


Figure S11: Bacterial community phylum-level contributions visualized by individual (“A”, “B”, and “C”) and body region (arm, foot, hand, leg, lower trunk, skull, tooth, upper trunk). Only relative abundances greater than 1% are displayed.
